# Using High-Throughput Animal or Cell-Based Models to Functionally Characterize GWAS Signals

**DOI:** 10.1007/s40142-018-0141-1

**Published:** 2018-05-29

**Authors:** Pierre Dourlen, Julien Chapuis, Jean-Charles Lambert

**Affiliations:** 1 INSERM U1167, RID-AGE-Risk Factors and Molecular Determinants of Aging-Related Diseases, Lille, France; 2 Institut Pasteur de Lille, Lille, France; 3 University Lille, U1167-Excellence Laboratory LabEx DISTALZ, Lille, France

**Keywords:** HCS, HTS, *Drosophila*, Screen, GWAS, Alzheimer

## Abstract

**Purpose of Review:**

The advent of genome-wide association studies (GWASs) constituted a breakthrough in our understanding of the genetic architecture of multifactorial diseases. For Alzheimer’s disease (AD), more than 20 risk loci have been identified. However, we are now facing three new challenges: (i) identifying the functional SNP or SNPs in each locus, (ii) identifying the causal gene(s) in each locus, and (iii) understanding these genes’ contribution to pathogenesis.

**Recent Findings:**

To address these issues and thus functionally characterize GWAS signals, a number of high-throughput strategies have been implemented in cell-based and whole-animal models. Here, we review high-throughput screening, high-content screening, and the use of the *Drosophila* model (primarily with reference to AD).

**Summary:**

We describe how these strategies have been successfully used to functionally characterize the genes in GWAS-defined risk loci. In the future, these strategies should help to translate GWAS data into knowledge and treatments.

## Introduction

Genome-wide association studies (GWASs) determine groups of single nucleotide polymorphisms (SNPs) in linkage disequilibrium and which are associated with a particular disease, trait, or phenotype. The most significantly associated SNP is usually not the causative SNP, and (by convention) the signal is assigned to the closest gene. However, the presence of several genes in a GWAS locus and complex linkage disequilibrium patterns involving the sentinel SNP may make it difficult or even impossible to determine which gene is responsible for the observed association. Hence, identifying the causative gene and one or more functional SNP are major challenges in the GWAS field. For instance, new technologies have been developed to tackle this limitation with the observation that 85–95% of the GWAS-associated SNPs are significantly enriched at cell-type-specific regulatory regions: massively parallel reporter assay (MPRA) allowed to screen thousands of variants in order to assess their impact on transcriptional activity [1]. A further difficulty (even when the risk gene is known) relates to the determination of how the causative genes are functionally involved in the disease process. Indeed, it can be difficult to establish a causal link on the basis of the literature data alone.

To take account of these limitations, gene enrichment pathway analyses have been developed from GWAS dataset. The main idea is that genes associated with the disease risk will be over-represented in specific pathways involved in the disease process. In Alzheimer’s disease (AD), these analyses have pointed to the immune response, the regulation of endocytosis, cholesterol transport, and protein ubiquitination [2]. However, it must be borne in mind that this type of analysis is subject to major limitations (in addition to methodological issues): for example, the defined canonical pathways are far from complete, and many numerous genes have pleiotropic functions and are thus nominated in many different pathways. It is also possible that a gene may have an unknown function with relevance to the pathophysiological context.

**Fig. 1:**
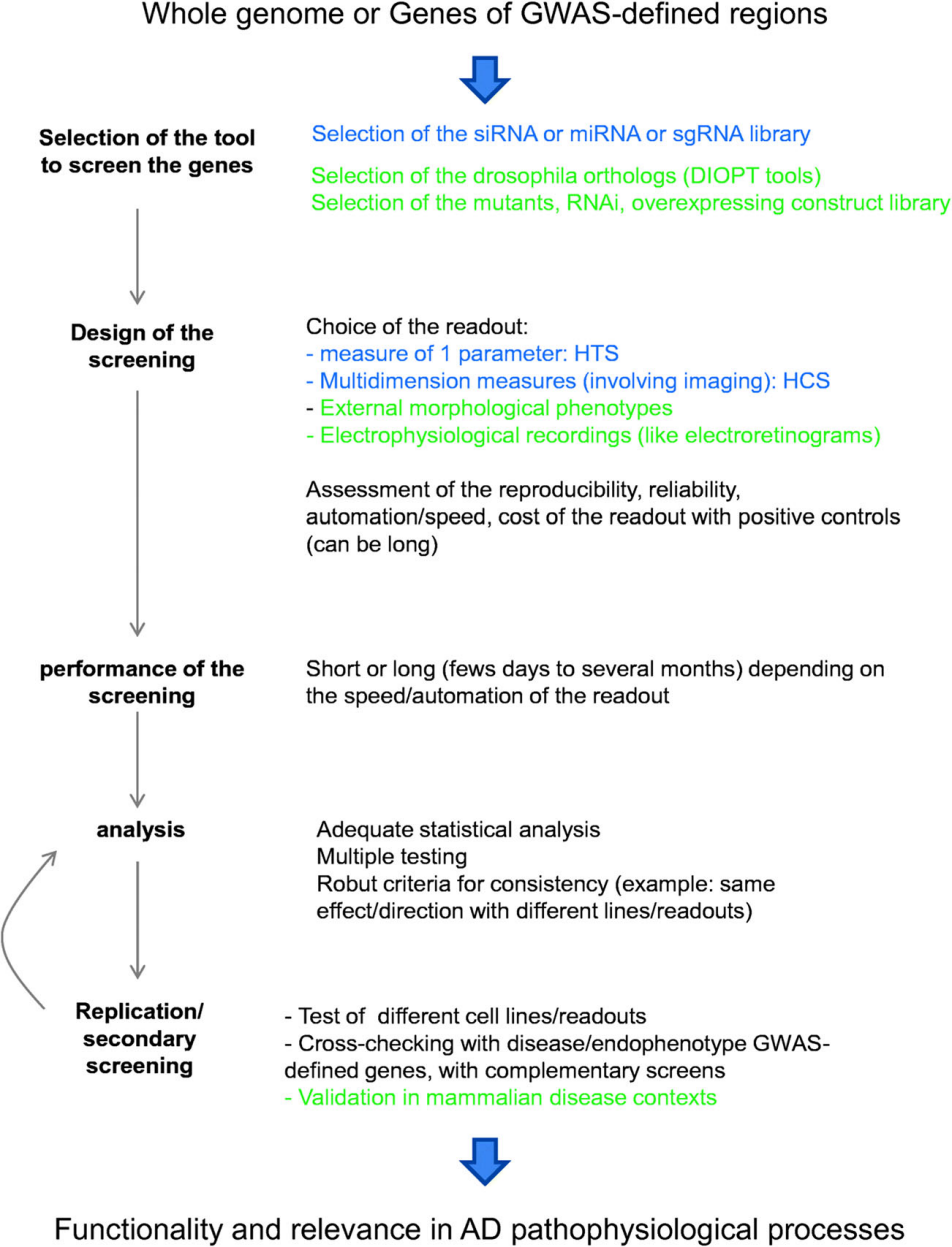
Schema describing the main steps to set up systematic screenings for GWAS-genomic data (in blue, specific to HCS/HTS; in green, specific to *Drosophila*)

Given this background, it appears that gene enrichment pathway approaches are not able to optimally exploit the genetic data generated by high-throughput genomic strategies. It is now possible to develop in silico methodologies by combining numerous datasets with the objective to define both functional variants and genes [3]. However, there is also a need for alternative, powerful approaches for empirically testing multiple GWAS genes in cell-based or animal models. Here, we review high-throughput functional screening strategies that use in vitro cell-based models and the in vivo *Drosophila* model for GWAS-defined hits (primarily in AD but also for some other neurological diseases) (Fig. 1). We will not cover *Caenorhabditis elegans*—another invertebrate animal model [4] that has also been used [5].

## High-Throughput Screening in Cell-Based Models

According to the amyloid cascade hypothesis (one of the main hypotheses in AD, in which the overproduction of amyloid (Aβ) peptides leads to neurotoxicity and then neuronal death), one expects at least some of the GWAS-defined genes to control the production of the Aβ peptides that accumulate in the AD brain. For decades, the impact of AD candidate genes on Aβ secretion/production was evaluated one by one. Given the large number of AD candidate highlighted by GWASs, these approaches no longer appear to be appropriate. Indeed, the assessment of a putative AD gene’s impact on Aβ peptides production requires cell-based models that are suitable for use in fast, large-scale screening.

One of the first attempts to systematically measure the impact of GWAS-defined genes involved the RNAi-mediated silencing of 24 late-onset AD genes in Hela cells stably over-expressing APP^sw^ [6]. The levels of APPs-β and Aβ_1–38/40/42_ secreted into the supernatant were then measured. Bali et al. proposed that late-onset AD genes did not specifically alter the Aβ42/40 ratio but probably contributed to AD through a distinct mechanism. However, this study was limited by the number of genes tested and the number of endophenotypes measured. Furthermore, the number of loci detected by GWAS is also increasing, meaning that this type of low-scale study is of less interest.

Technological progress has enabled the large-scale automation of biological experiments. These techniques are particularly appropriate for generating large amounts of data in cell-based models, and mark the entry of cell biology into the age of high-throughput methodologies (as has already happened for genetics, epigenomics, and transcriptomics, for example). Rather than focusing on subsets of genes, progress in genome annotation enables the systematic performance of high-throughput screening (HTS)—for instance by designing RNAi constructs to test the effects of gene silencing on biological phenotypes.

This type of systematic analysis was performed with 14,603 siRNA pools in HEK-293 cells stably expressing a mutant form of APP (NFEV) designed to enhance Aβ production. Conditioned media were used to quantify Aβ40, Aβ42, sAPPα, and sAPPβ levels [7, 8]. A “regulatory landscape of APP processing” was generated, in order to identify most of the pathways involved in the regulation of APP metabolism. Interestingly, some of the identified pathways contain genes (CLU, BIN1, CR1, PICALM, TREM2, SORL1, MEF2C, DSG2, EPH1A) linked to AD in the reference GWAS meta-analysis [9]. It is noteworthy that only the aggregate effect of genes in a pathway were considered as real effects, due to the high false-positive rate and the low level of reproducibility after siRNA library screening [10].

As mentioned above, assays of Aβ released in the cell culture medium have been used to identify modulators of APP metabolism. However, the underlying mechanisms controlling APP maturation and trafficking cannot be characterized solely by studying the amount of Aβ present in culture media. Thus, it was necessary to move to the high-content screening (HCS) of multidimensional phenotypes; this consists of the cell-based quantification of several processes simultaneously; the combination of cell-based and imaging methodologies provides a more detailed representation of the cell’s response to various perturbations than HTS does. In line with this approach, we developed a rapid, cell-based, HCS assay for the intracellular APP fragments in HEK293 cells stably over-expressing a mCherry-APP^695WT^-YFP construct [11]. The modified APP^695WT^ protein is known to be metabolized in the same way as APP^695WT^ [12]. Our model enabled the detection of specific APP products differentially tagged with mCherry or YFP (for the N- and C-terminal fragments, respectively). For instance, treatment with γ-secretase inhibitor was associated with a specific increase in YFP fluorescence intensity (corresponding to the APP’s C-terminal fragment). After customization for automatic image processing, we screened a genome-wide bank of 18,107 human siRNAs. In total, 832 hits were selected as being likely to have an impact on APP metabolism—including 8 genes associated with the risk of late-onset AD in the reference GWAS meta-analysis. These data suggested that the 8 genes are involved in AD process via the regulation of APP metabolism.

For HTS/HCS data, it is important to bear in mind that the choice of statistical methods used to define the initial hit list is critical; this will determine the relevant secondary analyses needed to narrow down the hits and thus investigate biological mechanisms. To assess the relevance of these genes with regard to Aβ peptide levels, we cross-checked our HCS data against an association study of SNPs and CSF Aβ42 peptide levels in a large sample of patients (*n* = 2950). Only SNPs within *FERMT2* were associated with low Aβ42 peptide levels; this highlighted FERMT2’s potential role in the AD process via the modulation of APP metabolism and Aβ peptide generation. Lastly, the impact of FERMT2 expression on APP metabolism was validated in several cell-based models, including a primary neuronal culture (PNC) endogenously expressing both APP and FERMT2. Therefore, accurate choice of statistical methods, crosschecking with other screens, and validation in low-throughput models enabled identifying FERMT2 as a risk factor modulating APP metabolism. The low number of final positive hits suggests that the AD genetic risk factors in the other loci may be involved in different processes than the APP metabolism. This is a limitation of the high-throughput methodologies. They are designed with respect to a defined phenotype. Most of the current models are based on neuronal dysfunctions involved in AD processes through APP metabolism and Aβ production. By using these approaches, it is not possible for example to investigate APOE or TREM2 functions which are thought to be mainly involved in Aβ clearance.

Other cell-based models compatible with HCS analysis have been generated to assess other phenotypes and could be used to test multiple GWAS genes—for instance QBI-293 cells with Dox-regulated inducible expression of human tau carrying the P301L mutation, and a GFP tag attached to visualize tau inclusions [13]. More recently, GFP bimolecular and trimolecular fluorescence complementation (biFC and triFC) has been used to study the localization and mechanisms of protein multimers (tau and TDP-43) in the context of neurodegeneration [14]. These technologies may enable new functional assays of genetic factors in neurodegenerative diseases through siRNA screening.

Some non-mammalian cell-based models like the *Drosophila* S2R+ cell line have also been used to perform unbiased, genome-wide screening of RNAi. *Drosophila* cell lines usefully have a low level of gene redundancy. RNA interference is also straight forward in these cells as dsRNA are added in the medium without transfecting reagent. This approach was exemplified by a screen for regulators of the translocation of Parkin (a protein whose mutation causes inherited recessive Parkinsonism) to mitochondria [15]. The genome-wide screen identified 60 genes, which were further narrowed down to 20 candidate genes with a conserved effect on both Parkin translocation and mitophagy in HeLa cells. The top hits belonged to the sterol regulatory element binding protein (SREBP)-lipogenesis pathway, including the master regulator of lipid synthesis sterol regulatory element binding transcription factor 1 (SREBF1). Interestingly, the latter is a GWAS-detected risk factor for sporadic PD suggesting a mechanistic link between inherited recessive PD and sporadic PD—a long debated question [16].

In order to explore many potential functions of the genetic risk factors, it is important to highlight the growing need to measure different phenotypes in different cell types. Indeed, gene expression that are cell-specific, like TREM2 (preferentially expressed in microglia), requests relevant and adapted cellular model. For this purpose, iPSC will likely play an important role in the future to generate multiple cell types like neurons, astrocytes, microglia, or endothelial cells susceptible to be involved in pathophysiological mechanisms. However, compared with easy-to-transfect standard cell lines, HCS approach using these iPSCs-derived cells will request viral transduction to modify gene expression and will thus lead to screen smaller number of genes in these different models for the moment. A lentiviral RNA interference library of 597 shRNAs was already used to screen for novel regulators of synapse formation [17]. In addition, the development of the CRISPR-CASS9 technology and sgRNA libraries should enable large-scale DNA editing and increase even more the power of HCS.

In conclusion, there is no doubt that HTS/HCS methodologies are very powerful ways of potentially assigning pathophysiological functions to GWAS-defined genes. It is likely that their use will increase over the coming years, although this requires major investments in dedicated, automated technology platforms. It is important to bear the following key issues in mind: (i) HTS/HCS models face many challenges, including the identification of the best experimental system and the development of robust, reproducible assays; (ii) HTS/HCS models are often highly sensitive, and can generate a large number of unspecific, biologically irrelevant responses; (iii) in view of the number of analyses to be performed, HTS/HCS approaches must balance the risk of observing significant results by chance against the risk of rejecting biologically valid hypotheses on purely statistical grounds; and (iv) as with all data generated in high-throughput assays, the most relevant observations need to be replicated and validated in other, unrelated cell-based models.

## *Drosophila* in Genetic Screening

Moving on from in vitro cell-based models, unbiased, high-throughput screens of GWAS-defined loci can be performed in a more integrated biological in vivo context by using relevant read-outs in animal models of the target disease. However, there are many obvious constraints associated with the use of animals and the inability to systematically screen hundreds of genes in a murine model of AD. This approach is nevertheless possible in small invertebrate models, such as the fruit fly *Drosophila melanogaster*. This fly shows good gene conservation with humans and only a little gene redundancy—enabling easier detection of the effects of loss of function. Two thirds of human disease-associated genes are estimated to have a functional homolog in *Drosophila* [18]. In contrast, gene regulatory regions are much less well conserved, therefore it is difficult to use *Drosophila* to identify a causative non-coding SNP if it affects gene expression. *Drosophila* has a short life cycle, with a new generation produced every 10 days at 25 °C. When the latter advantage is combined with high numbers of progeny and the low cost of housing, it is easy to produce the large number of individuals required for optimal genetic studies. Furthermore, many genetic tools have been developed in more than a century of use in forward and reverse genetics. One can therefore modulate more or less any genes, anywhere and at any point in the life cycle [19, 20]. Mutants are available for almost all the genes in the *Drosophila* genome [21]. Collections of RNA interference and overexpression constructs have also been generated at the genome-wide scale. Expression of these constructs is usually based on the Gal4/UAS system [22]. Modifications can make the system conditional, with expression controlled by changing the temperature (Gal80ts, temporal and regional gene expression targeting—TARGET—system, [23]) or by adding a drug to the flies’ food (the GeneSwitch system; [24, 25]). Thus, *Drosophila* offers many tools for tightly controlling gene expression. Furthermore, *Drosophila* has a wealth of external phenotypes (the size, color, and shape of the eye or of the wing, etc.) that are easily scorable under a dissecting microscope. This is essential for quickly screening the effect of gene modulation. Lastly, *Drosophila* has higher cognitive functions (like memory and sleep) for which behavioral and electrophysiological assays are available. These features make *Drosophila* an ideal model for neurogenetics.

## *Drosophila* for Screening GWAS-Defined Genes

In this context, *Drosophila* has been used to screen GWAS candidate genes associated with AD [26–28]. Starting from the reference GWAS meta-analysis [9], we screened for the AD candidate genes that modified tau neurotoxicity [28]. Based on the regional association plots, we identified a total of 148 genes in the 19 AD-associated loci. According to the *Drosophila* RNAi Screening Center Integrative Ortholog Prediction Tool (http://www.flyrnai.org/diopt; [29]), 54 of the human genes had a total of 74 *Drosophila* orthologs at 13 loci. Two hundred seventy-eight RNAi lines from five collections (the Japan NIG collection, the Vienna GD and KK collections, and the Harvard TRiP attP2 and attP40 collections) and 17 mutant and overexpression constructs were selected to modulate gene expression (giving four constructs per gene, on average). The fly eye was used to assess tau neurotoxicity. Expression of human tau (2N4R isoform) during *Drosophila* eye development results in small, rough eyes. Each of the 295 constructs was tested singly to see whether the size of the tau-expressing eye was modulated. After quantification of eye size, only genes for which at least two positive constructs from different collections had the same effect were considered to be positive hits. By applying these criteria, we confirmed that *Amph* (an ortholog of *BIN1*) modulated tau neurotoxicity [30]. We also identified four new genes: *p130CAS*, *Eph*, *Focal adhesion kinase* (*Fak*), and *Rab3-GEF*, which are respectively orthologs of *CASS4*, *EPHA1*, *PTK2B*, and *MADD*. Interestingly, three of the five hits (*Fak*, *p130CAS*, and *Eph*) are directly or indirectly involved in the cell adhesion pathway. Another *Drosophila* screen of tau neurotoxicity modifiers also identified genes involved in this pathway [26]. This is one of the advantages of unbiased systematic screening in *Drosophila*; the identification of new genes that modulate a process (tau neurotoxicity, in this case) and point toward a new pathway (the cell adhesion pathway, in this case) as being a potentially important pathway for AD pathogenesis. Of course, one can argue that the tau-associated fly eye phenotype reflects only one aspect of AD pathogenesis and the results does not explain how the hits are involved in the pathogenesis. Further work in *Drosophila* and mammalian models is required to address these questions. In our study, we were able to validate the genetic interaction between tau and *Fak* in a cell adhesion-related fly wing readout. By assessing tau phosphorylation and testing a catalytically mutant form of *Fak*, we could disfavor a change in tau phosphorylation (classically considered as one of the culprits in AD pathogenesis) as the cause of the modulation of tau neurotoxicity by *Fak* in *Drosophila*. A tau-PTK2B interaction was confirmed in the brains of a tau mouse model and human patients, since we observed an abnormal somatic accumulation of PTK2B with the appearance of tau oligomers and neurofibrillary tangles [28].

Along with unbiased medium- to high-throughput in vivo screening with easily scorable read-outs, *Drosophila* is also used to address more specific questions about the functionality of a gene with respect to the clinical and biological features of human diseases. The AD risk factor *PICALM* has been studied in *Drosophila*. Its ortholog in the fly (*Lap*) was shown to regulate autophagy and tau degradation [31]. PD is characterized clinically by motor and non-motor symptoms and histologically by dopaminergic neuronal loss and the formation of α-synuclein Lewy bodies. The gene coding for cyclin-G-associated kinase (*GAK*) was identified in a GWAS as a PD susceptibility gene. To analyze its PD-related functions, Song et al. turned to *Drosophila* and studied loss of function of the *Drosophila GAK* ortholog (*aux*) [32]. Knockdown of *aux* resulted in age-dependent locomotor impairment (in a climbing assay), a shorter lifespan, and progressive dopaminergic neuronal loss—as seen in α-Syn overexpression. Downregulation of *aux* also enhanced α-synuclein-induced dopaminergic neuronal death, and sensitized flies to the environmental toxin paraquat. All these phenotypes represent a broad spectrum of parkinsonian-like symptoms—supporting the idea that *GAK*/*aux* is a PD risk factor with a potential role in PD pathogenesis. Lifespan and the loss of dopaminergic neurons in *Drosophila* were also used to identify an interaction between the PD risk factors PARK16 and LRRK2 [33]. Another example of the use of *Drosophila* to assess disease-like functions is illustrated by studies of the *BTBD9* gene. The latter had been identified in a GWAS as a risk factor for restless legs syndrome (RLS), a sensorimotor neurological disorder characterized by (i) a compelling urge to move during periods of rest, (ii) relief with movement, (iii) involuntary movements in sleep, and (iv) fragmented sleep [34]. Functional analysis of *BTBD9* was first performed in *Drosophila* [35]. Loss of the *Drosophila* homolog *CG1826* (*dBTBD9*) markedly disrupted fly sleep, with concomitant increases in waking and motor activity. This study also showed that *dBTBD9* regulates brain dopamine levels, which is known to correlate with RLS expression in humans [35]. Even for behavior like hyperactivity or alcohol consumption, *Drosophila* has provided functional evidence to complement the sometimes rather correlative genetic data. In order to better understand dysfunctional reward processing and the discovery of an association between hyperactivity and the *VPS4A* gene in a GWAS in adolescents, a causal role of the sole *Vps4* ortholog for hyperactivity was validated in *Drosophila* [36]. Similarly, after the identification of the association of the *AUTS2* gene with alcohol consumption at a genome-wide level of significance, functional evidence was obtained in *Drosophila*; downregulation of the sole *AUTS2* homolog (*tay*) led to a lower alcohol sensitivity [37].

With the increasing use of nucleic acid sequencing in research and clinical practice, many rare coding variants with unknown functional consequences—called variants of unknown or uncertain significance, VUS—are being identified. *Drosophila* happens to be a very useful model for addressing this issue. The rationale consists in performing functional transcomplementation experiments in *Drosophila*, as successfully illustrated for VUS in the *TARDBP* gene [38]. *TARDBP* encodes the TDP-43 protein, which forms cytoplasmic inclusions in patients with the most frequent form of frontotemporal dementia (FTD) and most forms of amyotrophic lateral sclerosis (ALS) and in 60% of patients with AD. It is a common disease-causing factor in FTD and ALS. However, how *TARDBP* mutations cause neurodegeneration is not well known, especially with regard to their loss-of-function or toxic gain-of-function properties. Null mutants of the *Drosophila* ortholog of *TARDBP* (*TBPH*) lose the neurons in the ventral nerve cord that secrete the neurohormone bursicon [39]. It was shown that expression of human *TARDBP* in *TBPH*-null *Drosophila* rescued the bursicon neurons, thus indicating functional transcomplementation [38]. This made *Drosophila* a platform for testing TARDBP mutations. Expression of two typical ALS-causing mutations (p.G287S and p.A315T) could not rescue neuronal loss as efficiently as wild-type TARDBP did. One atypical variant (p.D169G) could rescue, and another (p.A90V) could not. These findings suggested a partial loss of function in TARDBP mutations [38]. The same strategy was successful for VUS in the *TM2D3* and *CACNA1A* genes, in the context of late-onset AD and ataxia [40, 41]. This method for assessing pathogenic properties of rare variants has been named “diagnostic strategy” and considered the third main approach to study human diseases using fly models, in addition to forward and reverse genetics [18].

## Conclusion

In the context of neurodegenerative disorders in general and AD in particular, high-throughput technologies appear to be very useful for characterizing the pathophysiological functions of GWAS-defined genes (Table 1). The next step will be likely to develop multidimensional high-throughput methods allowing to analyze at the same time both functional variants and gene functions in accurate cellular types and models. However, it is important to keep in mind that the more the study is complex, the more the statistical analyses and quality control are essential for the success of such sensitive methods to avoid false-positive/negative results. Furthermore, confirmation and validation in complementary low-throughput assays are systematically required. In addition, among practical issues, the high technicality of the methods can make them difficult to be mastered and cost effective in terms of equipments.

**Table 1:** Positive genes in high-throughput functional screening of AD GWAS-defined loci

Gene	GWAS locus	Functions	Hit in high-throughput screening
BIN1	BIN1	Nucleocytoplasmic adaptor protein involved in endocytosis and membrane recycling, cytoskeleton regulation, DNA repair, cell cycle progression, and cell death [42]	Modifier of tau (2N4R) toxicity in *Drosophila* eye [28]
CASS4	CASS4	Member of the CASS scaffolding protein localized at focal adhesions, regulates cell spreading and motility [43]	Modifier of tau (2N4R) toxicity in *Drosophila* eye [28]
CD2AP	CD2AP	Scaffolding protein involved in the regulation of membrane receptor endocytosis and signaling, actin cytoskeleton organization, endosomal vesicular trafficking, cell adhesion, and cytokinesis [44–49]	Modifier of tau (0N4R V337M) toxicity in *Drosophila* eye [26]
CELF1	CELF1	CUGBP Elav-like family member 1, role in RNA processing (splicing and mRNA stability mainly), role in myotonic dystrophy [50]	Modifier of tau (0N4R V337M) toxicity in *Drosophila* eye [26]
EPHA1	EPHA1	Founding member of the Eph family of tyrosine kinase receptor, Interaction with integrin-like kinase and regulation of cell morphology and motility through the ILK-RhoA-ROCK pathway, role of ephrin/EphR in synapse development and plasticity [51–53]	Modifier of tau (2N4R) toxicity in *Drosophila* eye [28]
FERMT2	FERMT2	Focal adhesion protein involved in integrin activation [54]	Modifier of tau (0N4R V337M) toxicity in *Drosophila* eye [26] modifier of APP metabolism by HCS [11]
MADD	CELF1	Rab3/Rab27 guanine nucleotide exchange factor, role in synaptic vesicle trafficking; interaction with TNF receptor, role in cell death/survival signaling [55, 56]	Modifier of tau (2N4R) toxicity in *Drosophila* eye [28]
PTK2B	PTK2B	Member of the focal adhesion kinase (FAK) family of protein tyrosine kinase. Role in signal transduction. Activated by neuronal depolarization, Ca2+, and stressful conditions, role in neurite outgrowth, in synaptic plasticity, in neuronal survival, in astrocyte mobility [57–62]	Modifier of tau (2N4R) toxicity in *Drosophila* eye [28]

Beyond these general comments, another limitation of the current high-throughput methods lies on the statement that these screens are based on processes already suspected to be involved in the disease, i.e., tau toxicity and APP metabolism in AD. This is efficient and useful to identify new actors of these processes and these actors may point toward a new pathway like the cell adhesion pathway (as observed in the screening we performed using tau toxicity in *Drosophila* eyes [28]). However, one can argue that the identification of novel and unexpected pathways and processes will be only achieved by developing models based on novel phenotypes. For example, with the identification of several genes involved in microglia [3, 9, 63], microglia activation will be likely a readout of high interest as already recently shown [64]. The advent of the DNA editing tools and iPSCs will enable the development of models always closer to the disease characteristics. In conclusion, even though it is essential to bear in mind biases and limitations, the systematic development of these methodologies in a variety of different models should enable us to (i) probe specific pathological events, (ii) build ever more complete databases, (iii) develop complex approaches based on systems biology, (iv) systematically assess the potential roles of new genes, and (v) define new therapeutic targets and treatments.
